# Association between the *FTO* A/T Polymorphism and Elite Athlete Status in Caucasian Swimmers

**DOI:** 10.3390/genes12050715

**Published:** 2021-05-11

**Authors:** Piotr Zmijewski, Agata Leońska-Duniec

**Affiliations:** 1 Jozef Pilsudski University of Physical Education in Warsaw, 00-809 Warsaw, Poland; 2 Faculty of Physical Education, Gdansk University of Physical Education and Sport, 80-336 Gdansk, Poland; leonska.duniec@gmail.com

**Keywords:** sport, swimming, body composition, performance

## Abstract

The *FTO* A/T polymorphism (rs9939609) has been strongly associated with body mass-related traits in nonathletic populations, but rarely with elite athletic performance. The aim of the study was to investigate the association between the A/T polymorphism and athlete status in elite swimmers. Polish swimmers (*n* = 196) who competed in national and international competition at short- (SDS; 50–200 m; *n* = 147) and long-distance events (LDS; ≥400 m; *n* = 49) were recruited. The control group included 379 unrelated, sedentary young participants. The participants were all Caucasians. Genotyping was carried out using real-time PCR. It was found that the chance of being an elite swimmer was lower in carriers of the AT and AA genotype compared with TT homozygotes (1.5 and 2.0 times, respectively). These findings were confirmed in an allelic association; the A allele was less frequent in the swimmers compared with controls (*p* = 0.004). However, when SDS were compared against LDS, no significant differences were observed in genotypic and allelic distribution. The results of our experiment suggest that the variation within the *FTO* gene can affect elite athlete status. It was demonstrated that harboring the T allele may be beneficial for achieving success in a sport such as swimming.

## 1. Introduction

In 2007, three independent studies showed that a cluster of single nucleotide polymorphisms (SNPs) in the first intron of the fat mass and obesity-associated gene (*FTO*) is strongly associated with body mass-related traits and predisposes to overweight and obesity among children and adults [1,2,3]. Recently, studies have confirmed that the *FTO* polymorphisms are highly significantly related to obesity-related traits such as body mass index (BMI), hip circumference, total body weight, body fat percentage, and cardiometabolic traits [4,5,6,7]. The observed associations are replicable across multiple ethnic populations as well as different age groups [8]. However, in populations of European or East Asian ancestry, the relationship between these SNPs is substantially stronger than in the African ancestry population [5].

The human *FTO* gene localized to the long arm of chromosome 16 (16q12.2) [1] is expressed in a wide range of human tissues, especially in hypothalamic regions of the brain, where control of food intake is centered [5,9]. It encodes a 2-oxoglutarate (2-OG) Fe (II) dependent demethylase [5]. In vitro studies have shown that this nuclear protein is able to remove methyl groups from 3-methylthymine in single-stranded DNA as well as 3-methyluracil and 6-methyladenosine in single-stranded RNA nucleotides. 2OG oxygenases require nonheme iron [Fe(II)] as a cofactor, use oxygen and, usually, 2OG as cosubstrates, and produce succinate and carbon dioxide as by-products [9]. It was suggested that potential roles for FTO are regulation of transcription of genes involved in metabolism and posttranslational modifications as well as repair and/or modification of nucleic acid by demethylation [5,9,10]. FTO can influence the activity of pathways controlling daily food intake, nutrient preference (intake of dietary fat and/or protein), appetite, and satiety as well as control overeating [5].

A common A/T polymorphism (rs9939609), located in the first intron of the *FTO* gene, is the most frequently examined SNP in the context of genetic predispositions for an increased risk of excess body weight in nonathletic populations. Considering the intronic location of the A/T polymorphism, it probably plays a transcriptional regulatory role, either to up- or downregulate FTO expression [5]. The A allele, defined as a risk allele, has been associated with the development of overweight and obesity, increasing the risk by 20–30%. About 16% of studied populations are homozygous for the risk alleles and these individuals weigh ~3 kg more than those not carrying the A allele [1]. 

Recently, some studies have suggested that the A/T polymorphism of the *FTO* gene is associated not only with an excess of human body weight gain due to an increase in adipose tissue, but also with greater lean mass (LM) independent of diet and physical activity. This may be evidence that the *FTO* genotype is linked to muscle properties [11,12,13]. A recent systematic review reported that polymorphisms in the *FTO* gene are strongly associated with enhanced susceptibility to excess body mass and they were identified as genetic variants linked to elite combat sports status [14]. It is well confirmed that anthropometrics and body composition are key parameters of physical performance and elite athletes vary phenotypically among events [15]. Long-distance events favor economy and endurance; consequently, the endurance of athletes has been attributed in part to their lower BMI and body fat. Conversely, short events favor strength and speed, so sprint athletes tend to have higher BMI and greater local muscle mass [15,16]. Therefore, Guilherme et al. assumed that genetic variants such as the *FTO* A/T polymorphism, which can influence body composition parameters, might affect elite athletic performance [14,16].

To date, only a few studies have attempted to explain the association between the *FTO* A/T polymorphism and sport predispositions. In a study comparing 1089 participants (530 elite rugby athletes and 559 non-athletes), Heffernan et al. suggested that the T allele is associated with increased LM and athlete status. Guilherme et al. confirmed that the *FTO* AA genotype is less frequent in Brazilian (*n* = 677) and Russian (*n* = 920) athletes more reliant on a lean phenotype and linked to a decreased proportion of slow-twitch muscle fibers, while it is over-represented in power and combat sport athletes of heavier weight categories [16]. However, Eynon et al. did not find an association between the A/T polymorphism and elite athlete status in a European Caucasian cohort including Spanish (*n* = 81), Polish (*n* = 214), and Russian (*n* = 256) athletes [17]. These inconsistent findings suggest that more replication studies are needed to establish the interaction between the *FTO* gene and athletic performance in different populations.

Therefore, the main aim of the present study was to assess the frequency distribution of the *FTO* A/T polymorphism (rs9939609) between short- and long-distance elite swimmers as well as a sedentary control group. We hypothesized that (1) the frequency of the A allele and the AA genotype would be lower among elite swimmers compared with control participants; (2) the frequency of the A allele and the AA genotype would be lower among long-distance athletes compared with other swimmers.

## 2. Materials and Methods

### 2.1. Ethics Statement

The Pomeranian Medical University Ethics Committee, Poland approved the study. The investigation protocols were conducted ethically according to the World Medical Association Declaration of Helsinki and to the Strengthening the Reporting of Genetic Association studies statement (STREGA). All participants were informed of the risks and benefits of the experimental protocols and a written consent form was completed by each participant or their parents if the subject was under 18 years of age. All personal information and results were anonymous.

### 2.2. Participants

One hundred and ninety-six Polish swimmers (104 males and 92 females; 20.31 ± 2.67 years) who competed in national and international competitions and achieved a result greater than 600 FINA points were recruited for the study. The level of 600 points differentiates well-trained athletes from amateurs (e.g., this score was required to achieve 10th place in females and 26th place in males, in the 200 m butterfly event at the 2013 National Championships). Mean swimming performance regarded as the personal best result was 734 points. To limit the confounding factors of health status, we chose only those athletes who obtained a Polish Swimming Federation license after the obligatory medical examination. All investigated swimmers had been finalists of the Polish National Championships. Additionally, seven of them had participated in the Olympic Games and 48 of them had taken part in the World Championships or European Championships. The whole group of swimmers included eight World Championship medalists, 15 European Championship medalists, and 128 Polish Championship medalists. The range of number of training units per week was 5–13. 

The swimmers were divided into two groups based on their competitive distance and values of relative contribution of the aerobic or anaerobic energy systems: long distance swimmers (LDS; *n* = 49; 24 males, 25 females) competing in 400 m or longer events and short distance swimmers (SDS; *n* = 147; 80 males, 67 females), competing at distances between 50 m and 200 m.

The control group included 379 (222 males and 157 females; 22.6 ± 2.8 years) unrelated, sedentary college students. The athletes and controls were all Caucasians to ensure that there was no ethnicity skew and to overcome any potential problems of population stratification. 

### 2.3. Genetic Analyses

DNA was extracted from the buccal cells using a GenElute Mammalian Genomic DNA Miniprep Kit (Sigma, Steinheim, Germany) according to the manufacturer’s protocol. All samples were genotyped in duplicate using an allelic discrimination assay on a C1000 Touch Thermal Cycler (Bio-Rad, Feldkirchen, Germany) instrument with TaqMan probes. To detect both FTO rs9939609 alleles, TaqMan Pre-Designed SNP Genotyping Assays (Applied Biosystems, Waltham, MA, USA) (assay ID: C__30090620_10) containing primers and fluorescently labelled (FAM and VIC) minor groove binder (MGB) probes were used.

### 2.4. Statistical Analyses

Hardy–Weinberg equilibrium expectations were compared with observed counts using the chi-square test with one degree of freedom. Allele frequencies were estimated using genotype counts. Genotype and allele distribution between groups were compared using the chi-square test. The models of inheritance (i.e., codominant, dominant, recessive, and overdominant) were constructed assuming a minor allele as the risk allele. Odds ratios with 95% confidence intervals (95% CI) were used as a measure of the strength of the association. All analyses were performed using STATISTICA version 13 (TIBCO Software Inc., http://statistica.io, accessed on 26 March 2021). *p* values of <0.05 were considered statistically significant.

## 3. Results

Genotype frequencies of the rs9939609 were in Hardy–Weinberg equilibrium in the controls (chi-square = 0.22, *p* = 0.639), SDS group (chi-square = 0.06, *p* = 0.806), and LDS group (chi-square = 0.22, *p* = 0.639). We found a significant difference in genotype (under the codominant and dominant models) and allele frequencies between swimmers and controls (Table 1). In general, under the codominant model, the chance of being a swimmer was lower in the carriers of the AT and AA genotype compared with TT homozygotes (1.5 and 2.0 times, respectively). Additionally, under the dominant model, the carriers of the A allele (AT + AA) had a lower chance of being a swimmer than the TT homozygotes (1.6 times). These findings were confirmed in an allelic association, where the A allele was less frequent in the swimmers compared with the controls (34.9 vs. 43.7, *p* = 0.004, OR 0.69, 95% CI 0.54–0.89). However, when SDS were compared against LDS, no significant differences were observed in genotypic and allelic distribution (Table 2), regardless of the model of inheritance.

## 4. Discussion

In the present study, we assessed the frequency distribution of the *FTO* A/T polymorphism (rs9939609) between SDS and LDS as well as a sedentary control group. The results confirmed the first hypothesis and showed that the frequencies of the AA and the AT genotype were lower among elite swimmers compared with the control participants (under the dominant and the codominant model). These findings were confirmed in an allelic association, where the A allele was less frequent in the athlete group. Therefore, we demonstrated that harboring the TT genotype may be favorable for achieving success in a sport such as swimming. These results confirmed that anthropometrics and body composition, influenced by the *FTO* A/T polymorphism, may be important parameters of elite athlete performance. However, the frequency of the AA and the AT genotype as well as the A allele did not differ between long-distance athletes and other swimmers, thus rejecting our second hypothesis.

In a previous study including 1089 Caucasian participants (530 elite rugby athletes and 559 nonathletes), Heffernan et al. suggested the relevance of the T allele and the TT genotype to increased LM, muscle-related phenotypes, and consequently, elite athletic status, compared to the A allele and the AA genotype. They reported greater T allele and TT genotype frequency in elite rugby union athletes playing positions more reliant on a lean phenotype for success, while the A allele and AA genotype were more common in positions where total body mass is more important [13]. These results were confirmed by Guilherme et al., who found that the A allele and the AA genotype were less frequent in Brazilian (*n* = 677) and Russian (*n* = 920) athletes more reliant on a lean phenotype such as long-distance athletes and combat sports athletes of lighter weight categories. Conversely, the A allele was over-represented in Russian power athletes and combat sports athletes of heavier weight categories. Moreover, they observed a lower proportion of slow-twitch muscle fibers in Russian athlete carriers of the AA genotype [17]. In a study of 42 rhythmic gymnastics involved in national and international events and in 42 control girls, 26.2% of athletes carried the *FTO* TT genotype in comparison to 7.1% of non-athletes [18]. These results confirm the observation in the present study of lower frequency of the A allele and the AA genotype among elite swimmers compared with the control group. On the other hand, in a study comparing 551 European athletes from Spain, Poland, and Russia and 1416 sedentary control participants, Eynon et al. did not find an association between the *FTO* genotype/allele frequencies and elite athletic status. They compared the endurance, sprint/power athletes, and controls with each other as well as comparing the groups of athletes with respect to the performance level, suggesting that the investigated genetic marker is not connected with elite athletic performance [16]. Another study conducted on the Polish population investigated whether the *FTO* A/T polymorphism influenced the effects of exercise training and obesity-related traits. An association was confirmed between the polymorphism and increased BMI in 201 young healthy women. However, the gene × physical activity interaction was not demonstrated [6]. Our results also confirmed that the *FTO* A/T polymorphism is a candidate marker for influencing body mass and body composition traits in a Caucasian population.

To date, a few possible biological mechanisms have been described underlying the association between the *FTO* A/T polymorphism and anthropometrics and, consequently, elite athletic performance. Emerging studies have shown that the associations between the intronic *FTO* polymorphisms and body composition are mediated through functional interactions with distal surrounding genes. The first intron includes a binding site for the transcriptional factor cut-like homeobox 1 (CUX1), which, through regulation of retinitis pigmentosa GTPase regulator interacting protein 1 (RPGRIP1L) expression, interacts with the leptin receptor and thereby affects food intake. In addition, this intron contains an enhancer sequence that directly binds to the promoter region of Iroquois homeobox 3 (IRX3) [4,13]. The *FTO* T allele is associated with decreased IRX3 expression compared to the A allele. This interaction may explain the greater LM through enhanced life-long motor neuron availability via oligodendrocyte transcription factor (OLIG2) expression and therefore may be a beneficial factor for certain forms of elite athletic ability and associated performance phenotypes [13]. Recently, associations between the *FTO* polymorphisms and insulin-like growth factor 1 (IGF-1)—specifically, serum IGF-1 levels were greater in T allele carriers—may also clarify the observed *FTO* genotype differences in body composition. IGF-1 is upregulated as a consequence of exercise/mechanical load and has a key role in skeletal muscle hypertrophy [19].

In many sport disciplines (e.g., running), short duration events promote strength and speed, while long duration events favor endurance and economy. As a consequence, elite runners differ phenotypically depending on the duration of the events. Indeed, anthropometrics of elite athletes are usually connected with speed and race distance, and the BMI/ground force generation ratio is constant across an event’s range. On the other hand, in swimming, the basic mechanical limitation on speed is the propulsive work performed per drag unit. It remains unclear whether the compromise in body composition also applies to swimmers. In the water environment, muscular effort to support body mass is minimal, since the body displaces water and is almost neutrally buoyant. However, an increased body and surface area will raise drag, which will reduce racing speed for a given amount of mechanical power. The studies examining the body composition parameters of swimmers have been inconsistent, with an unclear pattern of anthropometrics across sprint and endurance events [15,20,21]. Gagnon et al. analyzed data from the 2012 Olympics including 522 swimmers and 1060 runners to test whether runner and swimmer phenotypes varied across event distances. They found that across distances from the 50 m sprint to the 10,000 m marathon, elite swimmers converge on a single most favorable BMI in women’s and men’s events, in contrast to the strong inverse association between event distance and BMI in professional runners. These results have shown a key difference in design pressures and performance capability in terrestrial versus aquatic environments [15]. The conclusions put forward by Gagnon et al. may explain why the frequency of the A allele and the AA genotype did not differ between short- and long-distance swimmers in the present study.

A potential limitation of the present study is the relatively small group size of the elite swimmers, which might not show statistical power sufficient to yield meaningful analysis and interpretation of results. Additionally, unfortunately, anthropometric measurements were not collected, which made it impossible to complete the analysis. It needs to be highlighted that athlete phenotype is a polygenic trait. To date, over 155 genetic markers (located within 82 autosomal genes, mitochondrial DNA, X and Y chromosomes) have been linked to elite athlete status. To date, over 200 genetic markers have been linked to elite athlete status. They are called performance-enhancing gene polymorphisms (PEPs) and are located within autosomal genes, mitochondrial DNA, X and Y chromosomes. These include 93 endurance- and 69 power/strength-related genetic markers with a confirmed influence on the development of various sport-related traits [22,23,24] and could serve for usage of the total polygenetic scores, which could allow for the differentiation of power and endurance athletes [25]. Indeed, single-marker analysis is likely to make only a limited, but still important, contribution to understanding the athlete’s phenotype. 

## 5. Conclusions

The results of this study suggest that the variation within the *FTO* gene can affect elite athlete status. The observation of the lower frequency of the AA and the AT genotype as well as the A allele among elite swimmers constitutes the first important finding of the study, implying that some individuals may benefit from carrying the T allele. The carriers of the TT genotype had a 1.5–2.0 times higher chance of being an elite swimmer. These results support the hypothesis that anthropometrics and body composition, influenced by the *FTO* A/T polymorphism, are important for achieving success in sport. We also noted that the frequency of the A allele and the AA genotype did not differ in the study group of swimmers divided according to the length of the distance. The *FTO* gene was not found to be a strong and independent determinant of short-distance swimming performance. It is more likely that several gene loci, each with a small but significant contribution, are responsible for this genetic component.

## Figures and Tables

**Table 1:** Comparison of swimmers and control individuals (genotypes and alleles).

Genotype	Controls (*n* = 379)	SDS + LDS (*n* = 196)	OR (95% CI)	*p* †
	Codominant			
AA	70	24	0.49 (0.28–0.84)	0.015
	(18.5)	(12.2)		
AT	191	89	0.66 (0.45–0.97)	
	(50.4)	(45.4)		
TT	118	83	1	
	(31.1)	(42.4)		
	Dominant			
TT	118	83	1	0.008
	(31.1)	(42.4)		
AT-AA	261	113	0.62 (0.43–0.88)	
	(68.9)	(57.6)		
	Recessive			
TT-AT	309	172	1	0.051
	(81.5)	(87.8)		
AA	70	24	0.62 (0.37–1.02)	
	(18.5)	(12.2)		
	Overdominant			
AA-TT	188	107	1	0.257
	(49.6)	(54.6)		
AT	191	89	0.82 (0.58–1.16)	
	(50.4)	(45.4)		
A	331	137	0.69 (0.54–0.89)	0.004
	(43.7)	(34.9)		
T	427	255	1	
	(56.3)	(65.1)		

Note: OR—odds ratio (95% CI—confidence intervals), †—chi-square test.

**Table 2:** Comparison of SDS against LDS (genotypes and alleles).

Genotype	SDS (*n* = 147)	LDS (*n* = 49)	OR (95% CI)	*p* †
	Codominant			
AA	17	7	1.22 (0.44–3.34)	0.852
	(11.6)	(14.3)		
AT	68	21	0.91 (0.45–1.83)	
	(46.3)	(42.9)		
TT	62	21	1	
	(42.2)	(42.9)		
	Dominant			
TT	62	21	1	0.933
	(42.2)	(42.9)		
AT-AA	85	28	0.97 (0.51–1.87)	
	(57.8)	(57.1)		
	Recessive			
TT-AT	130	42	1	0.615
	(88.4)	(85.7)		
AA	17	7	1.27 (0.49–3.28)	
	(11.6)	(14.3)		
	Overdominant			
AA-TT	79	28	1	0.679
	(53.7)	(57.1)		
AT	68	21	0.87 (0.45–1.67)	
	(46.3)	(42.9)		
A	102	35	1.05 (0.65–1.69)	0.854
	(34.7)	(35.7)		
T	192	63	1	
	(65.3)	(64.3)		

Note: OR—odds ratio (95%CI—confidence intervals), †—chi-square test.

## Data Availability

The data presented in this study are available on request from the corresponding author. The data are not publicly available due to privacy/ethical restrictions.
